# Evaluating an Internet Gaming Disorder Scale Using Mokken Scaling Analysis

**DOI:** 10.3389/fpsyg.2019.00911

**Published:** 2019-04-26

**Authors:** Turi Reiten Finserås, Ståle Pallesen, Rune Aune Mentzoni, Elfrid Krossbakken, Daniel L. King, Helge Molde

**Affiliations:** ^1^Department of Clinical Psychology, University of Bergen, Bergen, Norway; ^2^Department of Psychosocial Science, University of Bergen, Bergen, Norway; ^3^School of Psychology, The University of Adelaide, Adelaide, SA, Australia

**Keywords:** Internet Gaming Disorder, pathological video gaming, psychometric properties, Mokken scale analysis, mental health

## Abstract

Internet Gaming Disorder (IGD) was recently included as a condition for further study in the fifth and latest version of the Diagnostic and Statistical Manual of Mental Disorders. The present study investigated whether the IGD criteria comprise a unidimensional construct. Data stemmed from a sample of Norwegians aged 17.5 years in 2012 and 19.5 years in 2014 (*N* = 1258). The study used the Mokken scale analysis to investigate whether the score of the different items on the IGD scale measured a single latent variable and if the scale functions differently for males and females. Correlation analysis was conducted between the scores on the IGD scale (count) and the Gaming Addiction Scale for Adolescents (GASA, categorical), both assessed in 2014. Negative binomial regression analyses were applied in order to investigate how different predictors of mental health assessed in 2012 were associated with IGD assessed in 2014. The Mokken scale analysis showed that all item-coefficients of homogeneity exceeded 0.3 when the whole sample completed the scale and when females completed the scale, indicating that the items reflect a single latent variable. In both cases moderate (*H* > 0.40) unidimensionality was shown. The item measuring “tolerance” did not exceed 0.3 in the scale when completed by males, indicating that only eight out of nine items reflect a single latent variable when applied to males only. The eight-item scale containing males showed weak (*H* > 0.30) unidimensionality. The correlation analysis showed a positive correlation between the scores on the IGD scale and the GASA (*r* = 0.71, *p* < 0.01) when assessed simultaneously and a positive but lower correlation (*r* = 0.48, *p* < 0.01) when assessed longitudinally. Results from the negative binomial regression analysis showed that previous video-game addiction, being male, depression, aggression and loneliness were significant predictors of IGD. The associations were small for all independent variables except previous video game addiction and gender where the associations were large. Although the results from the correlation analysis and regression analysis showed predictive validity of the scale, the results from the Mokken analysis suggest that the IGD scale may not be applied as a unidimensional scale when the tolerance item is included.

## Introduction

In 2013 the American Psychiatric Association (APA) included Internet Gaming Disorder (IGD) as a tentative disorder in Section III of the fifth edition of the Diagnostic and Statistical Manual of Mental Disorders (DSM-5; American Psychiatric Association [APA], 2013). Despite its specific name, the category refers to non-Internet video games as well, although these have been less researched (American Psychiatric Association [APA], 2013). Because IGD is a significantly important public health issue, more research on this topic is warranted, and more research is also required to determine whether IGD should be a formally included diagnosis in the DSM system (American Psychiatric Association [APA], 2013). Still, and notably, Gaming Disorder has been included in the 11th revision of the International Classification of Diseases (ICD-11; World Health Organization [WHO], 2018).

The DSM-5 lists nine IGD criteria reflecting the following symptoms: Preoccupation, tolerance, withdrawal, deception, escape, continuing despite problems, loss of control, giving up other activities, and negative consequences (American Psychiatric Association [APA], 2013). The cut-off for the proposed diagnosis is endorsement of five or more criteria, that is, a strict cut-off set so as to prevent over-diagnosis. We stress that research is needed to conclude whether IGD can be included in the DSM and whether these nine criteria individually constitute elements of a diagnosis. One step toward accomplishing this would be to examine if the IGD diagnostic criteria comprise a unidimensional construct.

Previous research has used different terminology to describe the phenomenon. This article uses “pathological video-gaming” in reference to studies conducted prior to IGD. Several different instruments assessing pathological video-gaming have been developed over the years but these can broadly be characterized as inconsistent (King et al., 2013). In a review of different instruments assessing pathological video-gaming, the Problem Videogame Playing Scale was concluded to provide the best overall measure of the suggested IGD diagnosis, while it was concluded that the adapted DSM-IV-TR pathological gambling criteria, the Game Addiction Scale for Adolescents (GASA) and the Young Internet Addiction Test provide the most relevant clinical information (King et al., 2013).

Several scales measuring IGD have recently been developed, among them a ten-item IGD test (IGD-10, Király et al., 2017), IGD short form (IGDS9, Pontes and Griffiths, 2016), and a long (27 items) and short form (nine items) of the IGD scale (Lemmens et al., 2015). One scale (IGD-20) has already been translated from English (Pontes et al., 2014) to Spanish (Fuster et al., 2016) and validated. In contrast to previous studies, the current study aimed to remain close to the wording from the IGD diagnostic criteria. Thus, the current study can enhance knowledge by examining the psychometric properties of the IGD diagnostic criteria. GASA was previously one of the most frequently used instruments to assess pathological video-gaming, as well as one of the measures that provide the most relevant clinical information (King et al., 2013), hence a substantial correlation between the GASA and a scale based on the new suggested diagnosis would support the convergent validity of IGD. Furthermore, previous studies have identified specific factors associated with IGD; hence, the same association to a tentative IGD scale would support the scales’ construct validity. In this regard it should be noted that studies have reported positive associations between IGD and being male (Ferguson et al., 2011; Brunborg et al., 2013; Wittek et al., 2015), depression (Mentzoni et al., 2011; Sarda et al., 2016), anxiety (Mentzoni et al., 2011), aggression (Lemmens et al., 2009), and loneliness (Lemmens et al., 2011). For depression, one study found a strong effect size when comparing non-gamers and problematic gamers (Mentzoni et al., 2011), while another study found depression to be the strongest predictor of IGD when controlling for academic performance and loneliness (Sarda et al., 2016).

The present study will contribute to the APA call for research by exploring the psychometric properties, concurrent and construct validity of the proposed IGD diagnostic criteria. This will be investigated by (1) examining if each of the IGD-criteria reflects a single latent trait, (2) exploring the correlation between the scores on the new IGD-criteria and the GASA, and (3) investigating whether previously identified correlates of pathological video gaming can predict scores on a new scale based on the IGD-criteria. We expected a high correlation between the IGD-scale and GASA in wave 3, and a lower correlation between the IGD-scale and GASA in wave 1. Furthermore, we expected previously identified correlates of pathological video gaming measured in wave 1 to predict IGD in wave 3. Because of the longitudinal nature of this study, the previously identified correlates of IGD can be identified as predictors instead of associations, an aspect that can strengthen the predictive validity of the new scale. A recent review found only 13 longitudinal studies on the topic of pathological video gaming (Mihara and Higuchi, 2017), which makes the present study one of the few longitudinal studies available on this topic.

## Materials and Methods

### Participants

Participants were assessed by means of a questionnaire in a three-wave (2012, 2013, and 2014) longitudinal study. Wave 1 comprised Norwegians aged 17.5 years who were in their second year in upper secondary school. Evry AS selected a random non-stratified sample from the National Population Registry of Norway. Initially, 3,000 adolescents were invited to participate, 1500 females and 1500 males. The response rates for waves 1, 2, and 3 were 70.5, 52.0, and 52.0% (of those initially invited to participate), respectively, and are in line with a suggested norm for response rates (Baruch, 1999). Participants were included in the final analysis only if they had answered all the criteria in the IGD scale in wave 3 (*N* = 1258); consequently, six people were excluded from the sample. In addition, one participant was excluded because of low age and 14 were excluded because of lacking information on gender.

### Procedure

Participants were able to answer the questionnaire on paper or online. Only the participants who answered the first wave were invited to participate in wave 2. In the third wave, participants who responded to wave 1 were again invited to participate. For all three waves, the questionnaire assessed pathological video-gaming, anxiety, depression, aggression, and loneliness. In addition, a scale explicitly based on the nine criteria for IGD listed in DSM-5 (American Psychiatric Association [APA], 2013) was included in wave 3. All participants provided written informed consent. The participants were informed that their answers would be treated confidentially, and that everyone who answered the questionnaire would receive a gift voucher worth 200 Norwegian Kroner (∼25 US$). Participants received a new gift voucher for answering wave 2 and again for wave 3.

### Measures

The Hospital Anxiety and Depression Scale was used to measure symptoms of depression and anxiety (Zigmond and Snaith, 1983). The scale has seven items reflecting depression and anxiety symptoms, respectively. Items are rated on a four-point scale ranging from 0 to 3. A composite score was computed for both subscales. Internal consistency (Cronbach’s alpha) in the current study was 0.69 (*n* = 1239) for depression and 0.77 (*n* = 1240) for anxiety.

The Buss-Perry Aggression Questionnaire (physical and verbal aggression subscales) was used to assess aggression (Diamond and Magaletta, 2006). The physical aggression subscale contains four items, while the verbal aggression subscale contains three items. All items are rated on a five-point scale (1 = very unlike me and 5 = very like me). Internal consistency (Cronbach’s alpha) for the two subscales combined in the current study was 0.81 (*n* = 1238).

The Roberts UCLA Loneliness Scale was used to measure loneliness (Roberts et al., 1993). The scale consists of eight items. Respondents registered their responses on a four-point scale (1 = never and 4 = often). Four of the items were reverse-coded. A composite score was computed by adding the participant’s responses on all items. Internal consistency (Cronbach’s alpha) for the scale in the current study was 0.77 (*n* = 1224).

The seven-item version of GASA (Lemmens et al., 2009) was used to assess pathological video-gaming in all three waves. Respondents were asked about their experiences with games over the last 6 months, and ranked their responses on a five-point scale (1 = never and 5 = very often). Internal consistency (Cronbach’s alpha) for the scale in the current study was 88 (*n* = 1248) for wave 1 and 0.88 (*n* = 1251) for wave 3. The respondents were first divided into four categories of gamers, namely addicted gamers, problem gamers, engaged gamers and normal gamers based on a procedure previously described (Brunborg et al., 2013; Brunborg et al., 2015). Respondents who indicated that symptoms assessed by the four items reflecting core components of addiction (relapse, withdrawal, conflict, and problems) had occurred at least “sometimes” (King et al., 2013) were classified as addicted. Respondents scoring at least “sometimes” (King et al., 2013) on two or three of the same items were classified as problem gamers. Respondents scoring at least 3 on the first three items reflecting peripheral symptoms (salience, tolerance, mood modification) and who did not score 3 or above on more than one of the core criteria items were classified as engaged. The remaining respondents were categorized as non-problem gamers. The respondents were thereby divided into two categories of gamers, namely addicted gamers in one group, and non-addicted gamers truncated into one group. Respondents who did not play games were included in the category of non-addicted gamers.

In Wave 3, IGD was assessed using a scale explicitly based on the nine new criteria for IGD listed in DSM-5 (henceforth called IGD scale, American Psychiatric Association [APA], 2013). Respondents indicated their answers as “yes” or “no” on all nine items, and were given the following instructions: “The questions below relate to your relationship with computer games played on the Internet during the last 12 months. Tick the option that best suits you.” Internal consistency (Cronbach’s alpha) for the scale in the current study was 0.78 (*n* = 1258). The clarity of the items was not verified; however, the wording of the self-report measure was adapted as closely as possibly from the formulations found in the DSM-5 (American Psychiatric Association [APA], 2013), while at the same time, striving for simple language. To translate the questionnaire into English a forward-backward translation was done by a professional English copy-editor and a professional Norwegian copy-editor. Table 1 shows the English translation of the self-report measure.

**TABLE 1:** The Internet Gaming Disorder scale (IGD scale).

1	Have you spent a lot of time thinking about games or planned gaming? (Preoccupation)
2	Did you get annoyed, uneasy or upset when you couldn’t play? (Withdrawal)
3	Have you felt the need to play more and more? (Tolerance)
4	Have you tried to cut down on gaming without succeeding? (Loss of control)
5	Have you lost interest in previous hobbies and leisure activities because of gaming? (Giving up other activities)
6	Did you continue to play even though it created problems for you? (Continuing despite problems)
7	Have you lied to family members, therapists or others about how much you have played? (Deception)
8	Did you play to reduce negative feelings (like helplessness, guilt, anxiety)? (Escape)
9	Have you risked or ruined an important relationship, job, education or career opportunity because of gaming? (Negative consequences)

### Statistical Analysis

Descriptive statistics of all variables were calculated. The Mokken scale analysis was used to investigate whether the score of the different items on the IGD scale reflected the same latent variable, in the whole sample and separately for males and females. Mokken scaling is a non-parametric item response model that is typically used for evaluating measurement scales in psychology (Molenaar and Sjitsma, 1984; Stochl et al., 2012). One assumption in Structural Equation Modeling and Rasch modeling is multivariate normality. In comparison, the Mokken analysis is much less stringent because it makes no assumption about the functional form of the relationship between a particular item and the latent trait. It only requires that the ICCs meet the assumptions of double monotonicity. Therefore, the Mokken model will prove superior as a test for unidimensionality in the case of items with widely different difficulty levels. Another feature of the double monotonicity is called invariant item ordering, which implies that the ordering of the items is the same at all locations on the latent measurement continuum. This enables researchers to order the items according to difficulty, and means that the endorsement of a difficult item implies the endorsement of less difficult items. The scalability of the scale is measured by Loevinger’s coefficient of homogeneity (*H*). The present study used the same cutoff values used in previous studies (Molenaar and Sjitsma, 1984; Stochl et al., 2012). All values of *H* should exceed 0.3 in a unidimensional scale. Values between 0.3 and 0.4 indicate low accuracy, 0.4 and 0.5 indicate medium accuracy, while values over 0.5 indicate strong accuracy (Stochl et al., 2012). Alpha was set to default, 0.05. There were no missing data in the Mokken scaling analysis.

Pearson correlation coefficients were calculated to investigate if the number of criteria endorsed on the IGD scale in wave 3 correlated with being male (male = 1) in wave 1, high scores on depression, anxiety, aggression and loneliness in wave 1, and the number of criteria endorsed on the GASA in waves 1 and 3, respectively.

Descriptive statistics for the IGD showed a non-normal distribution with a high zero-count. Because of this, a negative binomial regression analysis was conducted where the additive sum score of the IGD items comprised the dependent variable (assessed at wave 3), and where gender, depression, anxiety, aggression and loneliness (all assessed at wave 1) were included as predictors and entered simultaneously. The dichotomous GASA scale (1 = addicted gamers, 0 = non-addicted gamers) assessed at wave 1 was included to control for pathological video-gaming in wave 1. The quality of the answers was obtained by checking for randomness and extreme response. Missing data for the correlation analysis and regression analysis were treated by excluding cases listwise.

The statistical analysis was conducted using IBM SPSS Statistics, version 24 (IBM Corp, 2016); R, version 3.3.0 (R Development Core Team, 2016), was used for conducting the Mokken scale analysis with the R-package Mokken (Andries van der Ark, 2007) including new developments (Andries van der Ark, 2012).

## Results

The sample consisted of 481 males (38.2%) and 777 females (61.8%). Participants had on average 2.06 siblings (*SD* = 1.36) and had a grade average of 4.24 (*SD* = 0.72) on a scale from 1 to 6. Most of the sample (65%, *n* = 818) lived with both parents. In all, 45.2% (*n* = 568) of their mothers and 39.6% (*n* = 498) of their fathers had higher education. Participants played video games on average 1.28 h per day on weekdays (*SD* = 1.96) and 2.03 h per day on weekends (*SD* = 2.87). Females played video games on average 0.75 h per day on weekdays (SD = 1.69) and 1.14 h per day on weekends (*SD* = 2.26), while males played video games on average 2.13 h per day on weekdays (*SD* = 2.06) and 3.46 h per day on weekends (*SD* = 3.16). In all, 418 females (54.6%) and 44 males (9.3%) reported not playing video games. In all, 462 participants (37.3%) reported not playing video games.

Table 2 presents descriptive statistics for the instruments used in the present study. The depression and anxiety means were below clinical range, as expected in a representative sample of youths. In wave 3, using the IGD criteria resulted in a higher proportion of respondents being classified as addicted, compared to GASA (2.3 and 1%, respectively).

**TABLE 2:** Descriptive statistics for the instruments.

	Mean (SD)	Skew	Kurtosis	*N*
*HADS-A*	5.60 (3.6)	0.81	0.69	1240
Males	4.66 (3.05)	1.03	1.64	474
Females	6.19 (3.71)	0.65	0.35	766
*HADS-D*	3.56 (2.95)	1.22	1.74	1239
Males	3.60 (2.91)	1.33	2.46	473
Females	3.53 (2.98)	1.16	1.35	766
*Buss-Perry aggression questionnaire*	12.65 (4.88)	1.27	1.48	1238
Males	13.43 (4.85)	0.92	0.56	473
Females	12.17 (4.84)	1.54	2.39	765
*UCLA loneliness scale*	4.83 (3.98)	1.21	1.52	1224
Males	4.70 (3.82)	1.15	1.39	469
Females	4.92 (4.08)	1.24	1.55	755
*GASA Wave 1*	10.30 (4.61)	1.69	2.83	1248
Males	12.88 (5.21)	0.94	0.52	477
Females	8.70 (3.32)	2.81	10.31	771
*GASA Wave 3*	9.60 (4.21)	2.10	4.76	1251
Males	11.62 (4.90)	1.33	1.91	476
Females	8.36 (3.14)	3.23	12.13	775
*IGD-scale*	0.43 (1.15)	3.50	14.15	1258
Males	0.82 (1.50)	2.26	5.31	481
Females	0.20 (0.78)	5.84	42.75	777

*Details concerning the distributions of scores in GASA and the IGD scale can be found in Supplementary Tables S1, S2.*

Table 3 presents the results of the Mokken scaling analysis for items on the IGD scale. The scalability as measured by Loevinger’s coefficient of homogeneity (*H*) was 0.41 for the whole sample scale, which indicates medium accuracy, and 0.48 when completed by females. All item-coefficients of homogeneity exceeded 0.3 (item *H*) when completed by the whole sample and by females, indicating that the items reflect a single latent variable. When completed by males, item 3 did not exceed 0.3 and was thus removed from the analysis. The new analysis showed a scalability of 0.4, which indicates medium accuracy. Item 1 showed best fit (0.52) in the entire sample scale, when completed by females (0.55) and when completed by males (0.53), indicating strong accuracy, while items 3 (0.33) and 7 (0.32) fitted least well in the entire sample scale, indicating low accuracy. No items fell below 0.4 when completed by females, indicating that all items had medium or strong accuracy. There were no violations of monotonicity or invariant item ordering (IIO). The reliability of the Mokken scaling analysis is equivalent to that of classical test theory. The reliability was 0.78 for the whole sample, 0.76 when being completed by males and 0.80 when being completed by females.

**TABLE 3:** Mokken scaling analysis of Internet Gaming Disorder.

Item	Item description	Item *H* [95% CI]	Standard error of item *H*	Monotonicity means	Significant monotonicity violations	Significant IIO violations
1	Preoccupation	0.52 [0.44–0.6]***	0.04	1.09	0	0
	Males	0.53 [0.41–0.65]***	0.06	1.20	0	0
	Females	0.55 [0.41–0.69]***	0.07	1.03	0	0
2	Withdrawal	0.37 [0.29–0.45]*	0.04	1.03	0	0
	Males	0.36 [0.22–0.5]*	0.07	1.05	0	0
	Females	0.52 [0.36–0.68]***	0.08	1.03	0	0
3	Tolerance	0.33 [0.25–0.41]*	0.04	1.05	0	0
	Males^a^	–	–	–	–	–
	Females	0.45 [0.29–0.61]**	0.08	1.03	0	0
4	Loss of control	0.37 [0.27–0.47]*	0.05	1.03	0	0
	Males	0.36 [0.26–0.46]*	0.05	1.06	0	0
	Females	0.41 [0.17–0.65]**	0.12	1.01	0	0
5	Giving up other activities	0.34 [0.26–0.42]*	0.04	1.03	0	0
	Males	0.33 [0.23–0.43]*	0.05	1.07	0	0
	Females	0.42 [0.17–0.67]**	0.13	1.01	0	0
6	Continuing despite problems	0.47 [0.39–0.55]**	0.04	1.04	0	0
	Males	0.49 [0.39–0.59]**	0.05	1.09	0	0
	Females	0.53 [0.33–0.73]***	0.10	1.01	0	0
7	Deception	0.32 [0.24–0.4]*	0.04	1.03	0	0
	Males	0.33 [0.23–0.43]*	0.05	1.07	0	0
	Females	0.41 [0.1–0.72]**	0.16	1.01	0	0
8	Escape	0.43 [0.35–0.51]**	0.04	1.09	0	0
	Males	0.40 [0.3–0.5]**	0.05	1.15	0	0
	Females	0.51 [0.33–0.69]***	0.09	1.06	0	0
9	Negative consequences	0.40 [0.3–0.5]**	0.05	1.03	0	0
	Males	0.38 [0.28–0.48]*	0.05	1.06	0	0
	Females	0.49 [0.25–0.73]**	0.12	1.01	0	0

Item *H* = Loevinger’s coefficient; CI = confidence interval; IIO = invariant item ordering. *indicates low accuracy, **indicates medium accuracy, ***indicates strong accuracy. ^a^The tolerance item did not exceed 0.3 on item *H* in the scale when completed by males and was therefore removed from the analysis.

The results of the correlation analysis showed a moderate positive correlation between the scores on the IGD scale and the GASA assessed at wave 1 (*r* = 0.48, *p* < 0.01) and a large positive correlation between the scores on the IGD scale and the GASA assessed at wave 3 (*r* = 0.71, *p* < 0.01). Likewise, the IGD scale correlated positively with gender (male = 1, *r* = 0.26, *p* < 0.01), anxiety (*r* = 0.09, *p* < 0.01), depression (*r* = 0.23, *p* < 0.01), aggression (*r* = 0.14, *p* < 0.01), and loneliness (*r* = 0.2, *p* < 0.01), respectively. The correlation between the IGD scale and anxiety was trivial (*r* = 0.09, *p* < 0.01).

Table 4 presents the results of the negative binomial regression analysis and show that addicted gamers assessed with GASA at wave 1, gender, depression, aggression and loneliness were significant predictors of IGD. The full model was statistically significant (χ^2^ = 306.68, *df* = 6, *p* < 0.01).

**TABLE 4:** Negative binomial regression analysis where the sum score (range 0–9) of Internet Gaming Disorder comprised the dependent variable.

	*B*	*SE*	OR [95% CI]
Gender (a)	1.39	0.13	4.01 [3.12–5.15]**
Anxiety	0.02	0.02	1.02 [0.98–1.06]
Depression	0.09	0.02	1.09 [1.05–1.14]**
Aggression	0.03	0.01	1.03 [1.00–1.05]*
Loneliness	0.06	0.02	1.07 [1.03–1.10]**
GASA Wave 1	1.09	0.24	2.97 [1.85–4.77]**

SE = standard erros; OR = odds ratio; CI = confidence interval. *p < 0.05; **p < 0.01. (a) 1 = male, 0 = female. 1 Dispersion parameter = 1.17.

## Discussion

The aim of the present study was to investigate the psychometric properties of a new scale assessing IGD. The scores on the IGD scale showed a high correlation with another instrument of IGD, and associations with gender and small associations of categories of psychiatric distress were found to be in line with previous literature and thus demonstrate the predictive validity of the new scale. The results from the Mokken analysis indicate that one may not apply the IGD scale as a unidimensional scale when the tolerance item is included.

The results from the Mokken scaling analysis completed by males showed that one item did not exceed the limit of 0.3 on item *H*, and was thus excluded from the analysis. This was the item measuring “Tolerance” in the scale. One study on the tolerance item used open-ended questions and found that gamers increasingly desired game items, status or story progress, but none reported a need for increasing time spent gaming (King et al., 2017). Petry et al. (2014) suggested that the phrase “playing more exiting games or use more powerful equipment” should be added to the item. However, this has also been debated (Griffiths et al., 2016). Nonetheless, the results from the present study suggest that merely asking about the need to play is not valid to discriminate between gaming addiction and non-addiction. In the present study, the greater response rates by females may have influenced the results on the scale when analyzing responses from both genders together, where the tolerance item did exceed 0.3 on item *H*. Future research should apply the eight item scale of the IGD scale and the tolerance item should be reworded and tested further.

The monotonicity means stemming from the Mokken scale analysis reveal that the preoccupation criterion and the escape criterion are the easiest to endorse This is in line with Rehbein, Kliem, Baier, Mößle, and Petry (Rehbein et al., 2015), who found these two criteria were most often endorsed, although Rehbein et al. (2015) concluded that these items actually are not valid to discriminate between gaming addiction and non-addiction. Also, Király et al. (2017) reported these items as being less important than the others because the former items added little information to the estimation of IGD severity. The preoccupation criterion has been critically discussed previously (Griffiths et al., 2016). Griffiths et al. (2016) state that since gaming is a common pastime among children, adolescents and adults, being preoccupied with games is not necessarily indicative of problematic gaming. In contrast, the criterion of escape has been linked to problems with gaming in a number of studies (Billieux et al., 2011; Kuss et al., 2012). However, recent studies suggest that it is present at an equal rate in non-problem gamers and problem gamers alike, suggesting that it may not be indicative of problematic gaming in itself (Ko et al., 2014; Lemmens et al., 2015). In conclusion, there seems to be agreement consensus that the preoccupation criterion is one of the easiest items to endorse and that this item may not be indicative of problematic gaming. There is still disagreement regarding the escape criterion, although the present study supports the notion that it is easy to endorse. Future research should investigate this criterion further.

The prevalence of IGD was 2.3% in this study when assessed with the IGD scale. This is similar to previous research reporting a prevalence of 2.4% (Przybylski et al., 2017) and 2.9% (Király et al., 2017). In contrast, Rehbein et al. (2015) found a prevalence of 1.2%, which is considerably lower than the percentage reported in the present study. However, in the present study, we employed dichotomous response options (no/yes), whereas Rehbein et al. (2015) used a four-point scale and included two questions for each of the nine criteria. Endorsing a criterion in line with that approach implied responding “strongly agree” to one of the two questions reflecting a specific criterion. A previous Norwegian population study found a prevalence of 1.4% (Wittek et al., 2015). However, participants in that study were between 16 and 74 years old, while the present study’s prevalence rate was based on 19.5 year olds. As young age is associated with IGD (Wittek et al., 2015), a higher prevalence would be expected in the present study’s population. Another Norwegian study of adolescents with a mean age of 13.6 reported a prevalence of 4.2% (Brunborg et al., 2013). Petry et al. (2014) also found a lower prevalence (0.3–1.0%) when adding a significant distress criterion. In line with this, Carras and Kardefelt-Winther identified a group who scored high on symptoms for IGD, but did not score high on other problems (Colder Carras and Kardefelt-Winther, 2018). This indicates that prevalence rates might be elevated when distress is not taken into account. The World Health Organization has notably added a functional impairment requirement to the criteria of Gaming Disorder because of the importance of this (World Health Organization [WHO], 2018).

Depression, aggression and loneliness were all found to be positively associated with IGD in the current study, although the effect sizes were small. This is in line with previous research (Lemmens et al., 2009; Lemmens et al., 2011; Mentzoni et al., 2011) and supports the predictive validity of the IGD scale. The present study found similar small correlations between aggression and IGD as a previous study (Lemmens et al., 2009), and the same small effect size for loneliness as a previous study (Lemmens et al., 2011). Gender was included in the regression model in the present study, and explains most of the variance in the model. This might explain why we found a smaller effect size for depression than a previous study which did not include gender (Mentzoni et al., 2011). The effects sizes for the categories of psychiatric distress was small in the present study relative to gender, which questions how meaningful these categories are in predicting IGD in a youth sample. This is in line with a recent study which concluded that the association between digital technology use and adolescent well-being is negative but small, explaining at most 0.4% of the variation in well-being (Orben and Przybylski, 2019). In line with findings reported by Sarda et al. (2016), we found a significant correlation between anxiety and IGD score, but this relationship was not significant in the regression analysis. It should be noted that one study actually reported a negative relationship between IGD and anxiety, which may reflect that anxiety may be lowered by operating in a predictable world of games (Andreassen et al., 2016). In terms of future studies it should be noted that the use of negative binomial regression implies that no transformation is needed to get from the regression parameters on the right-hand side of the equation to the normal distribution.

### Strengths and Limitations

The present study demonstrates a number of strengths. By using a large sample randomly selected from the national population registry, the results can be generalized across the population. However, because of the young sample in the current study, the results may not be generalized to other age groups without reservations. Further research should examine the validity of the IGD scale in different subpopulations. Another strength of the present study is the 2-year gap between data collection of the predictors and the dependent variable, which shows relationships over time, as opposed to pure cross-sectional studies, and makes this one of the few longitudinal studies on IGD.

Because of the exclusive reliance on self-report measures, however, the present study suffers from well-known biases like recall bias, social desirability bias and so on. Cronbach’s alpha was low for the HADS-D in the present study (0.69). Although a score >0.7 is suggested as acceptable for short scales with less than ten items (Pallant, 2013), HADS has been validated by several studies (Bjelland et al., 2002). In addition, the studies we have compared our results to have used HADS to assess predictors (Mentzoni et al., 2011; Sarda et al., 2016). Therefore, we chose to include depression in the analysis. Furthermore, the scale in the current study asked participants to consider only games played over the Internet. However, in the DSM-5, the supporting text specifies that offline games are included as well. This might have lowered the diagnosis percentage of IGD in the current study, as well as influenced the comparison to GASA, as offline games were not excluded there. In addition, the supporting text in the DSM-5 specifies that endorsement of five or more items is indicative of significant impairment or distress. It can in this respect be argued that this does not correspond to endorsement of the criteria based on the GASA, especially the polythetic approach, which implies that at least four of the seven items need to be answered “sometimes” or more frequent. However, in the present study categorization of addicted gamers based on GASA emphasized only core symptoms and excluded engaged gamers as well as problem gamers from this category. It is thus conceivable that respondents in the addicted category experienced concomitant distress. Still, the correspondence between the GASA-categorization and the IGD-scale and the experience of significant impairment or distress should be investigated in future studies. Furthermore, comparing the IGD scale to a measurement instrument other than GASA might have yielded different results. Another limitation is the low follow-up rate in this study. Finally, more females responded than men. The reason for this might be that females in general respond to surveys more often than men do, which is also true among students (Sax et al., 2003; Porter and Whitcomb, 2005).

## Conclusion

The present study reported results where the IGD scale correlates with a previous measure of IGD, the GASA. Compared to females, males had four times the odds of having at least one more IGD symptom. The odds of having at least one more IGD symptom increased by 9% for every one unit change in depression, 3% for every one unit change in aggression, and 7% for every one unit change in loneliness. These associations were shown to be in line with previous literature and therefore demonstrate predictive validity of the scale. Although the results from the correlation analysis and regression analysis showed predictive validity of the scale, the results from the Mokken analysis suggest that one may not apply the IGD scale as a unidimensional scale when the tolerance item is included.

## Ethics Statement

The study procedures were carried out in accordance with the Declaration of Helsinki and the Norwegian Health Research Act. The study was approved by the Regional Committee for Medical and Health Related Research Ethics in South East Norway (No. 2012/914). All participants were informed about the study in writing and provided informed consent.

## Author Contributions

All authors listed have made a substantial, direct and intellectual contribution to the work, and approved it for publication.

## Conflict of Interest Statement

The authors declare that the research was conducted in the absence of any commercial or financial relationships that could be construed as a potential conflict of interest.
