# Supplementary material for: Evaluating an Internet Gaming Disorder Scale Using Mokken Scaling Analysis
Source: Front Psychol. 2019 Apr 26;10:911. doi: 10.3389/fpsyg.2019.00911 (PMC6497737; doi:10.3389/fpsyg.2019.00911)
Supplement: Supplementary file 1 [file Table_1.docx]

**Supplementary Table S1**

*Distribution of scores in the IGD scale*

| Item | Not endorsed | Endorsed |
| --- | --- | --- |
| 1. Preoccupation | 1139 (90.5%) | 119 (9.5%) |
| 2. Withdrawal | 1215 (96.6%) | 43 (3.4%) |
| 3. Tolerance | 1195 (95%) | 63 (5.0%) |
| 4. Loss of control | 1217 (96.7%) | 41 (3.3%) |
| 5. Giving up other activities | 1219 (96.9%) | 39 (3.1%) |
| 6. Continuing despite problems | 1206 (95.9%) | 52 (4.1%) |
| 7. Deception | 1218 (96.8%) | 40 (3.2%) |
| 8. Escape | 1143 (90.9%) | 115 (9.1%) |
| 9. Negative consequences | 1225 (97.4%) | 33 (2.6%) |
