# Supplementary material for: Evaluating an Internet Gaming Disorder Scale Using Mokken Scaling Analysis
Source: Front Psychol. 2019 Apr 26;10:911. doi: 10.3389/fpsyg.2019.00911 (PMC6497737; doi:10.3389/fpsyg.2019.00911)
Supplement: Supplementary file 2 [file Table_2.docx]

**Supplementary Table S2**

*Distribution of scores in GASA wave 1 and wave 3*

|  | Never | Almost never | Sometimes | Often | Very often | N |
| --- | --- | --- | --- | --- | --- | --- |
| Wave 1 |  |  |  |  |  |  |
| 1. Salience | 827 (65.9%) | 231 (18.4%) | 123 (9.8%) | 49 (3.9%) | 24 (1.9%) | 1254 |
| 2. Tolerance | 736 (58.7%) | 248 (19.8%) | 183 (14.6%) | 69 (5.5%) | 18 (1.4%) | 1254 |
| 3. Mood modification | 821 (65.5%) | 187 (14.9%) | 151 (12.0%) | 70 (5.6%) | 25 (2.0%) | 1254 |
| 4. Withdrawal | 873 (69.8%) | 182 (14.5%) | 134 (10.7%) | 53 (4.2%) | 9 (.7%) | 1251 |
| 5. Relapse | 1065 (85.1%) | 123 (9.8%) | 46 (3.7%) | 10 (.8%) | 8 (.6%) | 1252 |
| 6. Conflict | 1045 (83.5%) | 121 (9.7%) | 60 (4.8%) | 20 (1.6%) | 5 (.4%) | 1251 |
| 7. Problems | 959 (76.6%) | 159 (12.7%) | 95 (7.6%) | 29 (2.3%) | 10 (.8%) | 1252 |
| Wave 2 |  |  |  |  |  |  |
| 1. Salience | 914 (72.7%) | 181 (14.4%) | 112 (8.9%) | 39 (3.1%) | 11 (.9%) | 1257 |
| 2. Tolerance | 847 (67.4%) | 200 (15.9%) | 155 (12.3%) | 43 (3.4%) | 12 (1.0%) | 1257 |
| 3. Mood modification | 858 (68.3%) | 167 (13.3%) | 140 (11.1%) | 60 (4.8%) | 31 (2.5%) | 1256 |
| 4. Withdrawal | 993 (79.2%) | 147 (11.7%) | 82 (6.5%) | 16 (1.3%) | 16 (1.3%) | 1254 |
| 5. Relapse | 1128 (89.7%) | 77 (6.1%) | 32 (2.5%) | 17 (1.4%) | 3 (.2%) | 1257 |
| 6. Conflict | 1126 (89.6%) | 76 (6.1%) | 44 (3.5%) | 7 (.6%) | 3 (.2%) | 1256 |
| 7. Problems | 978 (77.9%) | 148 (11.8%) | 94 (7.5%) | 23 (1.8%) | 12 (1.0%) | 1255 |
